# Achieving High Thermal Conductivity in Epoxy Composites: Effect of Boron Nitride Particle Size and Matrix-Filler Interface

**DOI:** 10.3390/polym11071156

**Published:** 2019-07-06

**Authors:** Sasan Moradi, Yolanda Calventus, Frida Román, John M Hutchinson

**Affiliations:** Departament de Màquines i Motors Tèrmics, ESEIAAT, Universitat Politècnica de Catalunya, C/Colom 11, 08222 Terrassa, Spain

**Keywords:** epoxy, thiol, boron nitride, thermal conductivity, differential scanning calorimetry (DSC)

## Abstract

For the thermal management of high watt density circuit layers, it is common to use a filled epoxy system to provide an electrically insulating but thermally conducting bond to a metal substrate. An epoxy-thiol system filled with boron nitride (BN), in the form of 2, 30 and 180 µm platelets, has been investigated with a view to achieving enhanced thermal conductivity. The effect of BN content on the cure reaction kinetics has been studied by differential scanning calorimetry and the thermal conductivity of the cured samples has been measured by the Transient Hot Bridge method. The heat of reaction and the glass transition temperature of the fully cured samples are both independent of the BN content, but the cure reaction kinetics is systematically affected by both BN content and particle size. These results can be correlated with the thermal conductivity of the cured systems, which is found to increase with both BN content and particle size. For a given BN content, the thermal conductivity found here is significantly higher than most others reported in the literature; this effect is attributed to a Lewis acid-base interaction between filler and matrix.

## 1. Introduction

Composite systems consisting of epoxy resin filled with boron nitride (BN), or other suitable fillers such as aluminium nitride or silicon carbide, are widely used for the thermal management of electronic devices on account of their high thermal conductivity and electrical insulating properties, combined with ease of manufacture. There are several commercial systems available with thermal conductivities of approximately 3.0 W/mK, for example VT-4B3 from Ventec [1] and Cool-CladTM from AI Technology [2], while even higher values can be found, though these often require some special preparation procedures. However, given the very high thermal conductivity of the boron nitride, up to 600 W/mK parallel to the basal plane and 30 W/mK perpendicular to the basal plane for hexagonal BN [3,4], one might expect that values considerably higher than 3.0 W/mK could be achieved with relatively simple preparation procedures. Such an outcome, though, is generally not observed, and there have been numerous studies aimed at understanding how the thermal conductivity depends on the epoxy-BN composite fabrication process, and what are the important parameters involved; these aspects are discussed in some recent reviews of thermal conductivity in polymer-based composites [5,6].

The BN content in the epoxy matrix composite is clearly of prime importance, and a systematic increase of thermal conductivity as the BN content increases is almost universally observed, the rate of increase accelerating for higher BN contents [7,8]. However, there is a practical limit to the BN content, as the viscosity of the simple epoxy-BN mixture increases rapidly and the mixture becomes essentially intractable for contents greater than 40 to 50 vol %; to achieve higher vol % contents requires the use of solvents or other procedures.

Besides this basic parameter of BN content, though, there are other parameters which play an important role in determining the thermal conductivity of the composite. These other parameters include the use or otherwise of surface treatments of the particles or of coupling agents and, in particular in the present context, the size and shape of the BN particles. As regards these last parameters, there is no universal consensus about the effect of particle size on the thermal conductivity, although the large majority of workers find that the thermal conductivity increases with BN particle size e.g., [9,10,11,12,13,14]. This correlation is somewhat confounded, however, by the fact that the BN particle shape is not always the same in these studies. For example, Huang et al. [12] compare the performance of small spherical BN particles, 0.2 to 0.4 µm in size, to larger “flakes”, or platelets, with sizes in the range 3 to 6 µm, while Gaska et al. [13] compare 13 µm platelets with 25 µm agglomerates. In both cases the larger particles give higher thermal conductivities, but the question remains of whether this is a consequence of the particle size or of the particle shape.

On the other hand, some authors report higher thermal conductivities for composites filled with smaller BN particles [15,16], while Wattanakul et al. [17] report that higher mixing speeds and sonication, which both have the effect of reducing the original agglomerate size of 300 µm, give composites with higher thermal conductivity. Indeed, these authors state that “small particles generally give higher thermal conductivity than large particles due to the formation of greater number of conductive pathways”, citing Zhou et al. [18] with reference to silicone rubber filled with BN particles. To complete the picture, Permal et al. [19] report that, for epoxy composites filled with either 1 or 5 µm BN particles, there is no significant effect of particle size, though their values of thermal conductivity are very low for the rather high content (40 vol %) of BN used.

All of the above discussion is further complicated by the possible effects of coupling agents or of surface treatments of the BN particles, which were used in all except three [12,13,17] of the references cited in the above paragraphs. In summary, therefore, there is no clear conclusion that can be drawn about the effect of BN particle size on the thermal conductivity of epoxy-BN composites. The purpose of the present work is to clarify this situation by fabricating epoxy-BN composites filled with BN particles in the form of platelets covering a wide range of sizes, and without the use of any coupling agents or surface treatments. The thermal conductivity of the cured composites is measured, and in parallel we study the cure kinetics by differential scanning calorimetry (DSC), which has been shown earlier [7,8] to mirror, in a consistent and systematic way, the dependence of the thermal conductivity on BN content.

## 2. Materials and Methods 

### 2.1. Materials

The epoxy resin was a commercial diglycidyl ether of bisphenol-A, DGEBA (Araldite GY240, Huntsman Advanced Materials, Salt Lake City, UT, USA), with a nominal molecular weight per epoxy equivalent (eq) of 182 g/eq and a density of 1.17 g/cm^3^. The cross-linking agent was a thiol, pentaerythritol tetrakis (3-mercaptopropionate) (Sigma-Aldrich, Saint Louis, MO, USA), with a molecular weight of 488.66 g/mol and a density of 1.28 g/cm^3^. The thiol reacts with the epoxy in a “click” mechanism, with the formation of hydroxyl and thioether groups in a single step [20,21]. The curing reaction was initiated using a latent initiator, encapsulated imidazole LC-80 (Technicure, A&C Catalysts, Linden, NJ, USA), in the form of a powder, which was added in the proportion of 2 parts per hundred resin (phr).

The boron nitride (BN) particles were obtained commercially (Saint-Gobain Boron Nitride, Amherst, NY, USA), and were of different sizes within the same product category of CarboTherm platelets. The average platelet size, maximum particle size, tap density and specific surface area, as given in the manufacturer’s literature [22] for the three types of BN particles used here, were as follows:

PCTP2: 2 µm, 10 µm, 0.2 g/cm^3^, 10 m^2^/g

PCTP30: 30 µm, 100 µm, 0.6 g/cm^3^, 1 m^2^/g

PCTP30D: 180 µm, 1600 µm, 0.6 g/cm^3^, 1 m^2^/g

**Figure 1:**
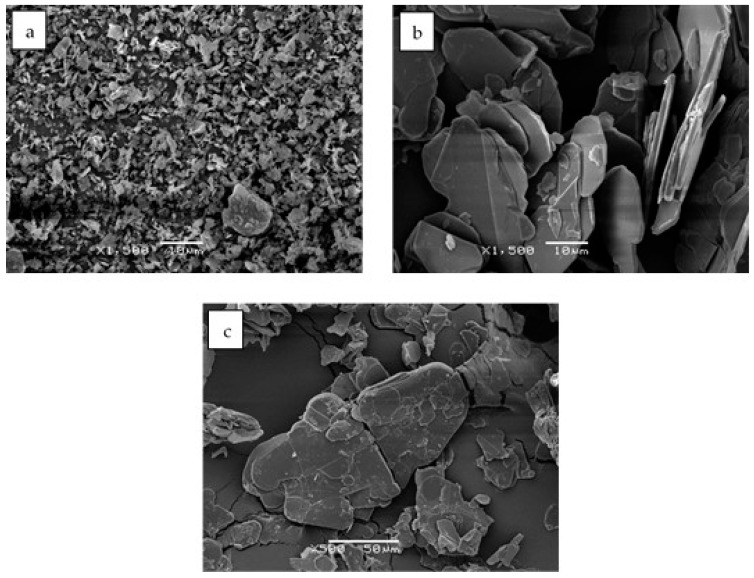
Scanning Electron Microscopy (SEM) images of the as-received BN platelet particles: (**a**) PCTP2, 2 µm, magnification 1500×, scale bar 10 µm; (**b**) PCTP30, 30 µm, magnification 1500×, scale bar 10 µm; (**c**) PCTP30D, 180 µm, magnification 500×, scale bar 50 µm.

Figure 1 shows the BN platelets of three different sizes in the as-received condition. Figure 1a,b, at the same magnification of 1500×, clearly show both the individual platelet characteristic of the 2 and 30 µm particles, respectively, and the significant difference in average size. There is inevitably a distribution of particle sizes, and according to the manufacturer’s information [22] the maximum particle sizes are 10 µm for PCTP2 and 100 µm for PCTP30. For example, in Figure 1a, a particle of 10 µm can be seen, and likewise, for PCTP30, particles as large as 100 µm have been observed.

The PCTP30D particles have been engineered for high shear mixing processes, for example for their incorporation into thermoplastics and, while being classified as platelets [22], they are slightly different from the other two platelets used here. These particles are stated by the manufacturer to be a “loosely agglomerated powder”, and there is a much wider distribution of particle sizes, with a maximum of 1600 µm. At the center of the image in Figure 1c, for example, there is a large (broken) particle, about 180 µm in the largest dimension, surrounded by much smaller particles.

### 2.2. Methods

#### 2.2.1. Sample Preparation

The epoxy and thiol were mixed by hand in a stoichiometric ratio, approximately 60:40 by weight, the latent initiator LC-80 previously having been added in the proportion of 2 phr. The BN particles were then added in the proportion required, to give contents of 10%, 30%, 50%, 60% and 70% with respect to the combined weight of epoxy and BN, and again were mixed by hand for approximately 10 min until a homogeneous mixture was obtained. This final mixture was then degassed in a vacuum chamber at room temperature and less than 26 hPa pressure for approximately 15 min. The sample denominations and proportions of all the components in each sample are given in Table 1.

The viscosity of the epoxy-thiol-BN mixture increases strongly with increasing BN content, and the mixture becomes a very stiff paste at the highest BN content. This is particularly so for the smallest BN particles (2 µm), for which the 70% BN content could not be achieved, because for the same BN content, the surface area to volume ratio increases as the particle size decreases. In fact, the specific surface area quoted by the manufacturer is ten times larger for PCTP2 than it is for the other two particle types. Furthermore, it is not possible to degas the samples with such high BN contents, and as a consequence it is likely that some air bubbles remain, which would tend to reduce the thermal conductivity. The preparation of these composites by this simple mixing technique therefore implies a limit to the BN content, which for the 70% samples represents a volume percentage of just less than 45%. To achieve higher vol % loadings of BN, as have been reported in the literature e.g., [10,14,23,24], involves more elaborate preparation procedures, such as the use of solvents, allowing filler contents as large as 80 vol % to be attained [10,14]. Even then, though, it is not uncommon to find that the thermal conductivity decreases beyond 50 or 60 vol %, probably because of the existence of voids.

#### 2.2.2. Differential Scanning Calorimetry (DSC)

DSC in both isothermal and non-isothermal (constant heating rate) modes was used to characterize the cure kinetics of these composite systems. The DSC instrument (Mettler-Toledo DCS821e, Greifensee, Switzerland) equipped with a robot sample handler and intracooler (Haake EK90/MT, Vreden, Germany), was calibrated for both heat flow and temperature using indium, and a flow of dry nitrogen at 50 mL/min was used throughout. The data analysis was made using the STARe software of the instrument.

For the isothermal cure experiments, the composite samples of about 10 mg, enclosed in an aluminium pan crimped with a lid, were weighed on an analytical balance and then immediately introduced into the DSC furnace, previously heated to the required isothermal cure temperature. The heat flow was measured as a function of time until the cure reaction was complete, the cure time depending on the cure temperature; cure temperatures of 60, 70 and 80 °C were used, all of which are sufficiently high temperatures such that no vitrification will occur during cure. The cure kinetics is characterized by the time, *t*_p_, at which the exothermic heat flow is a maximum, and by the exothermic heat of reaction, determined from the area under the cure curve. Subsequently, a second (non-isothermal) scan was made at 10 K/min to determine the glass transition temperature of the fully cured sample, *T*_g∞_, and to check that there was no residual heat of reaction. This second scan is made immediately after cooling at 20 K/min, and hence the glass transition temperature can be determined in the standard way as the mid-point of the transition in the specific heat capacity on heating from the glassy to the rubbery state.

For the non-isothermal cure experiments, the weighed samples were introduced into the DSC at 25 °C, cooled at 20 K/min to −65 °C, and then scanned at the required rate (2, 5 and 10 K/min) to 200 °C. The cure kinetics is characterized by the temperature, *T*_p_, at which the exothermic heat flow is a maximum, and by the exothermic heat of reaction, determined from the area under the cure curve. Subsequently, a second (non-isothermal) scan was made at 10 K/min to determine the glass transition temperature of the fully cured sample, *T*_g∞_.

#### 2.2.3. Thermogravimetric Analysis (TGA)

The TGA experiments were performed in a combined TGA/DSC (Mettler-Toledo TGA/DSC1, Greifensee, Switzerland), equipped with a robot sample handler and a cryostat (Huber ministat 125, Offenburg, Germany). The equipment was calibrated using indium with a dry air flow of 200 mL/min. For the TGA experiments, samples of 5 to 10 mg were heated at rates of 2 and 10 K/min from 40 to 600 °C with a dry nitrogen flow of 200 mL/min.

#### 2.2.4. Thermal Conductivity

The thermal conductivity was measured using the Transient Hot Bridge method (Linseis GmbH, THB-100, Selb, Germany), in which a heat pulse is applied to a sensor placed between two surfaces of the sample material and the resulting heat dissipation, quantified by the temperature change, Δ*T*, is used to determine the thermal conductivity directly [25]. The Kapton Hot Point sensor was calibrated with five standards, covering a range of thermal conductivities from 0.2 to 10 W/mK: polymethyl methacrylate, borosilicate crown glass, marble, a Ti-Al alloy, and titanium. The epoxy-BN composite samples were prepared by casting the uncured mixture into a silicone mold, 10 mm × 40 mm × 4 mm, degassing under vacuum at room temperature, and curing isothermally at 70 °C for 1 h. The surfaces of the cured samples were carefully polished by hand, using emery paper (120, 400 and 600 grit size sequentially), to give smooth and flat surfaces for contact with the Hot Point sensor. For the measurement, the sensor was clamped between these two smooth and flat surfaces, in a manual screw-actuated press, and a series of four repeated measurements were made, each with a heating power of 50 mW and a duration of 100 s, and allowing sufficient time between each measurement for the sample to return to the same initial state. The linear range of a plot of Δ*T* as a function of the inverse square root of the measurement time is extrapolated to zero on the abscissa (infinite time), and the thermal conductivity is then inversely proportional to Δ*T* (∞).

#### 2.2.5. Density

The density of the cured samples, ρ_s_, was determined by Archimedes principle, the samples being the same as those used for the thermal conductivity measurements. The weight of the sample in air, *m*_air_, and the weight of the same sample suspended by a fine wire in ethanol, *m*_eth_, for which the density, ρ_eth_, is tabulated as a function of temperature, were determined using an analytical balance. The sample density is calculated as:

ρ_s_ = *m*_air_ /(*m*_air_ − *m*_eth_) × ρ_eth_ (1)

#### 2.2.6. Scanning Electron Microscopy (SEM)

Fully cured samples, similar to those used for the thermal conductivity measurements and prepared using the same isothermal curing procedure, were fractured and then the fracture surface was sputter-coated with gold (Baltec SC-005, Leica Biosystems, Wetzlar, Germany) and examined in a Scanning Electron Microscope (JEOL JSM-5610, Tokyo, Japan). Typically, an accelerating voltage of 10 kV was used to give magnifications of 100× to 5000×.

## 3. Results and Discussion

### 3.1. Differential Scanning Calorimetry (DSC)

**Figure 2:**
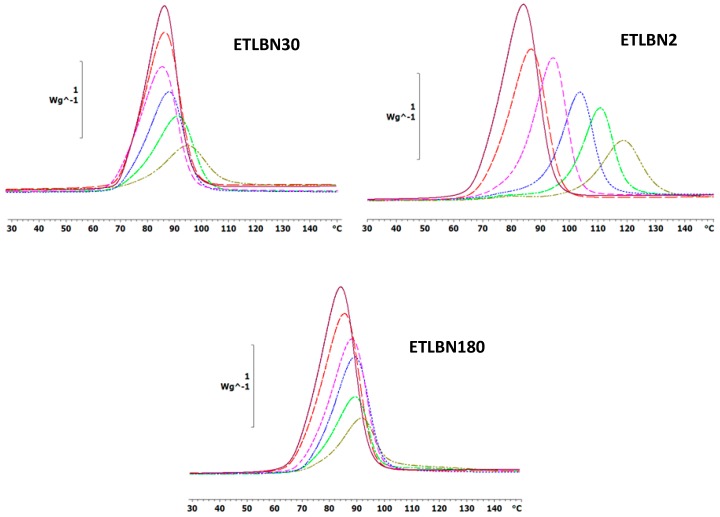
Non-isothermal DSC scans at 5 K/min for ETLBN2-*y*, ETLBN30-*y* and ETLBN180-*y*, in which *y* refers to the different BN contents given in Table 1: purple, full line, *y* = 0; long dash, red, *y* = 10; short dash, pink, *y* = 30; dash-dot, blue, *y* = 50; dotted, light green, *y* = 60; dash-double dot, dark green, *y* = 70.

The non-isothermal DSC curves at a heating rate of 5 K/min for the epoxy-thiol system without any filler (ETL) and for the epoxy-thiol-BN composite systems filled with 2, 30 and 180 µm BN platelets are shown in Figure 2, while similar results were obtained for heating rates of 2 and 10 K/min. These DSC scans were made from −60 °C, but only the temperature region in which the exothermic cure reaction occurs is shown here. At lower temperatures, however, the glass transition temperatures of the uncured mixtures, *T*_g0_, with 2, 30 and 180 µm BN platelets are observed at the average temperatures (±1 standard deviation) of −38.6 ± 0.5, −38.0 ± 0.7 and −37.5 ± 0.7 °C, respectively, compared with −38.4 ± 0.5 °C for the unfilled ETL system. The glass transition temperature of the uncured mixture is therefore essentially independent of the heating rate and of the BN content; the small increase in *T*_g0_ with increasing BN particle size may be a result of an increased restriction to the molecular mobility of the epoxy-thiol mixture with the larger particles, and is of no further consequence here.

In a second scan after the non-isothermal cure shown in Figure 2, the glass transition temperature of the fully cured system, *T*_g__∞_, is determined, and is found to be approximately independent of BN particle size and content, with values of 54.0 ± 0.4, 53.7 ± 1.2 and 52.7 ± 1.3 °C for the 2, 30 and 180 µm BN platelets, respectively. Likewise, the heat of reaction, Δ*H*, is also found to be essentially independent of BN particle size and content, as well as of the heating rate, with an average value of 127.2 ± 8.3 kJ/ee. The constancy of both *T*_g__∞_ and Δ*H* implies that the network structure of the cured epoxy composites is not influenced by the presence of the BN filler.

On the other hand, the kinetics of the cure reaction clearly is influenced by the BN filler, as can be seen from two particular aspects of the cure curves in Figure 2. The first aspect is the displacement of the curves as a function of BN content. The addition of BN particles results in a shift of the curves to higher temperatures. This is consistently observed for all BN platelet sizes, but is most dramatic for the smallest particle size of 2 μm, for which the exothermic peak is shifted by as much as 30 °C. The explanation for this observation probably lies in the relationship between surface area and volume of the BN platelets. If, as we argue here, there is an interaction between the epoxy-thiol matrix and the BN filler, which is a surface effect, then the surface area to volume ratio will have an important influence. In fact, it is probably only the edge surfaces of the platelets which are involved in this interaction, for which the area to volume ratio is inversely proportional to the particle size. Furthermore, the 180 µm platelets have a much wider distribution of particle sizes than do the other platelet sizes (see Figure 1), and there is also a tendency for the larger platelet sizes to break during fabrication, evidenced by the fracture surfaces of the cured composites (to be discussed below). Consequently, the area-to-volume ratio for the 30 and 180 µm platelets is rather similar, whereas that for the 2 µm platelets is an order of magnitude larger.

The second aspect is that the cure curves become increasingly asymmetric as the BN content increases, this being particularly noticeable in Figure 2 for the composites filled with 30 and 180 μm platelets. For the composites filled with the 2 µm platelets, on the other hand, this asymmetry takes the appearance of a shoulder at low temperatures, which becomes more prominent as the BN content increases, eventually even being manifest as a small peak for the highest BN content. The explanation for this behavior probably lies in the action of the LC-80 initiator, which is an encapsulated imidazole. This encapsulation means that, in principle, the initiation of the epoxy-thiol reaction by the LC-80 occurs when the temperature of 70 to 80 °C is attained during non-isothermal cure. In addition, though, the reaction kinetics is influenced also by the presence of the BN platelets, and in a different way depending on the platelet size, as discussed immediately above. The consequence of this combination of effects is the different asymmetry observed in Figure 2 for the 2 µm platelets in comparison with that observed for the 30 and 180 µm platelets.

**Figure 3:**
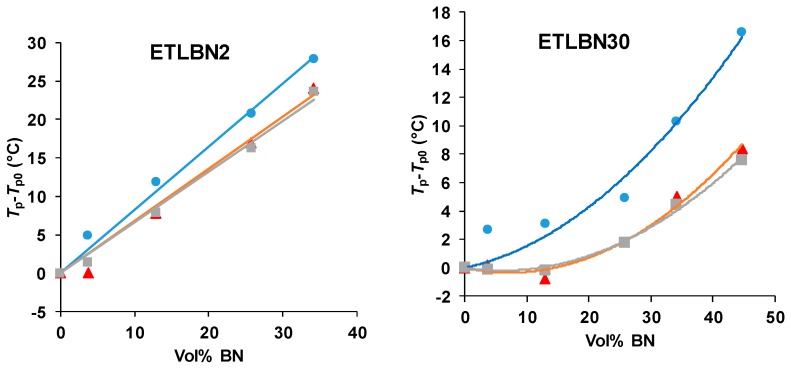

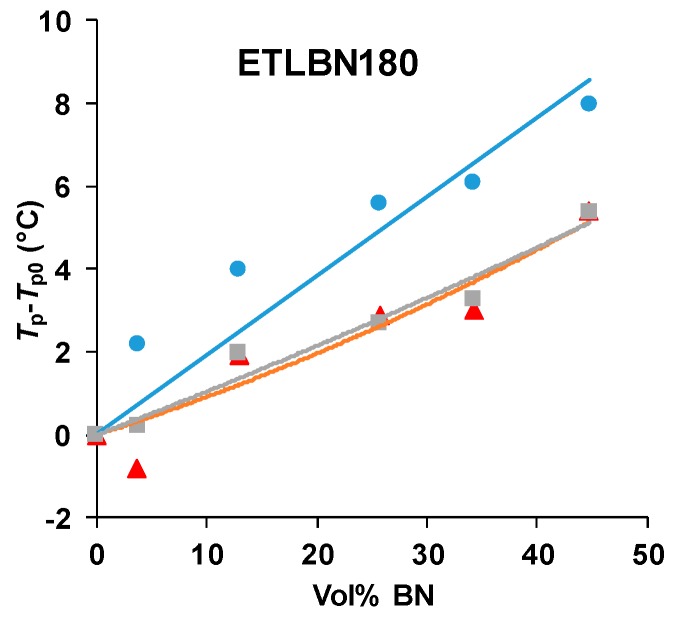
Peak temperature during non-isothermal cure relative to that for the unfilled system, *T*_p_−*T*_p0_, as a function of BN content for different platelet sizes, as indicated, and for different heating rates: 10 K/min, blue circles; 5 K/min, red triangles; 2 K/min, grey squares.

The shift of the peaks to higher temperatures with increasing BN content can be seen more readily in Figure 3, where the peak exothermic temperature, *T*_p_, relative to that for the unfilled system, *T*_p0_, is plotted as a function of the vol % of BN for each particle size and for each heating rate. The temperature shift is clearly greatest for the 2 µm platelets, and then reduces in turn for the 30 and 180 µm particles.

For all particle sizes, it is interesting to observe that there is no significant difference between the shifts for the two lower heating rates of 2 and 5 K/min. At first sight, this may appear strange, but it must be remembered that it is the *difference* between the peak temperatures of the filled and unfilled systems that is plotted in Figure 3; in all cases, the peak temperature itself always increases with increasing heating rate, as would be expected. What the results for the heating rates of 2 and 5 K/min probably indicate is that the cure reaction is not simple, as has already been commented in respect of the results presented in Figure 2, and that there may be competing effects of the BN filler particles. In particular, there appears to be a tendency, especially for the 30 µm composites, for the reaction first to be accelerated on the addition of BN particles, before being slowed.

**Figure 4:**
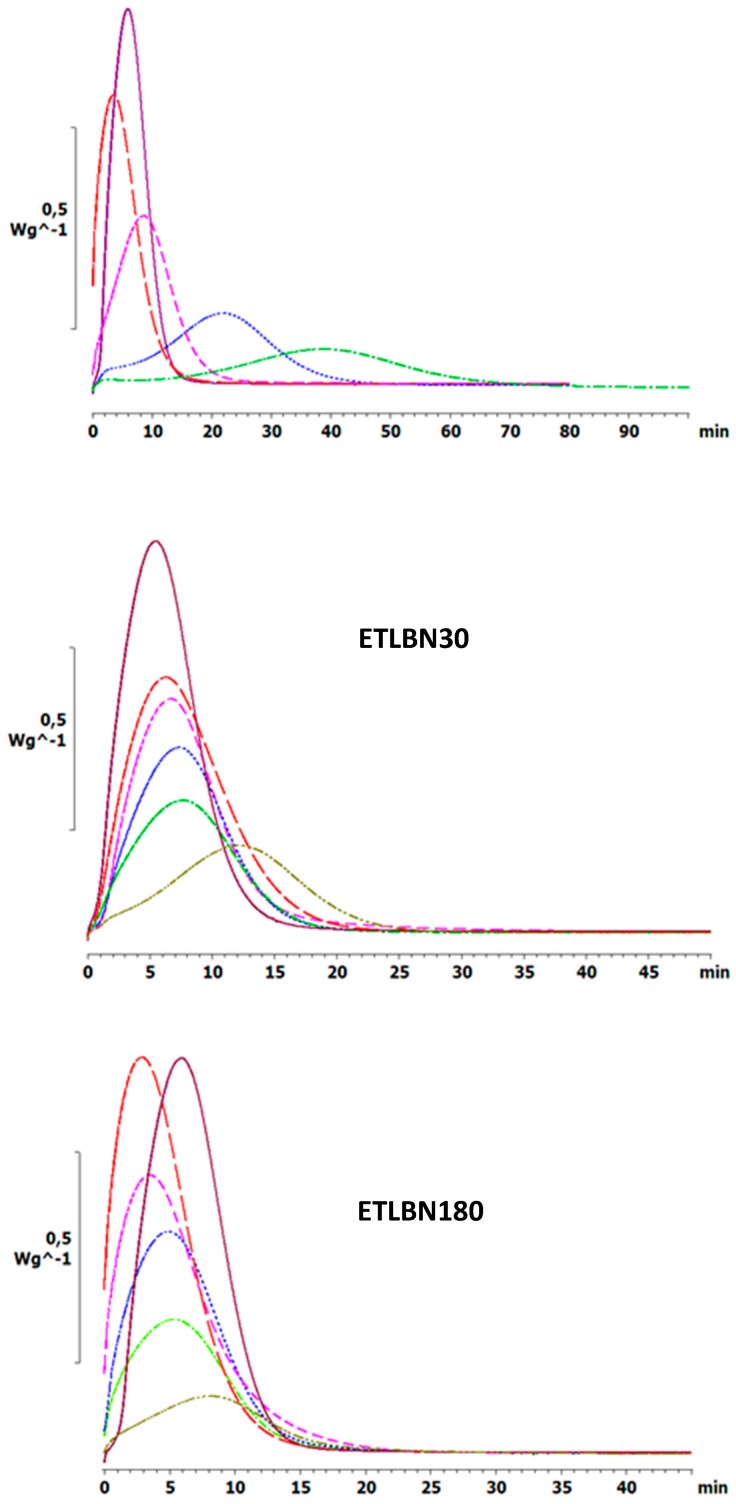
Isothermal DSC scans at 70 °C for ETLBN2-*y*, ETLBN30-*y* and ETLBN180-*y*, in which *y* refers to the different BN contents given in Table 1: purple, full line, *y* = 0; long dash, red, *y* = 10; short dash, pink, *y* = 30; dash-dot, blue, *y* = 50; dotted, light green, *y* = 60; dash-double dot, dark green, *y* = 70.

Similar effects are observed also for the isothermal cure. Figure 4 shows the isothermal cure at 70 °C of samples with different contents of the three BN particle sizes: ETLBN2, ETLBN30 and ETLBN180. For each cure condition, the heat of reaction (122.8 ± 9.6 kJ/ee) and the glass transition temperature of the fully cured sample (52.7 ± 0.8 for ETLBN2, 53.0 ± 0.9 for ETLBN30, 51.8 ± 1.3 °C for ETLBN180), obtained from a second scan, are essentially independent of the filler content and of the isothermal cure temperature. On the other hand, the cure kinetics is significantly influenced by the BN content. In general, with increasing amount of filler, whatever the size of the platelets, the exothermic peak is displaced to longer times, similar to the displacement to higher peak temperatures in the non-isothermal cure curves in Figure 2. However, for the smallest BN content (*y*=10), the cure is actually accelerated for the 2 and 180 μm platelets, an effect that can be observed also in some of the non-isothermal results, for example for the 30 µm platelets, shown in Figure 3. Similar results were found for the other isothermal cure temperatures of 60 and 80 °C.

**Figure 5:**
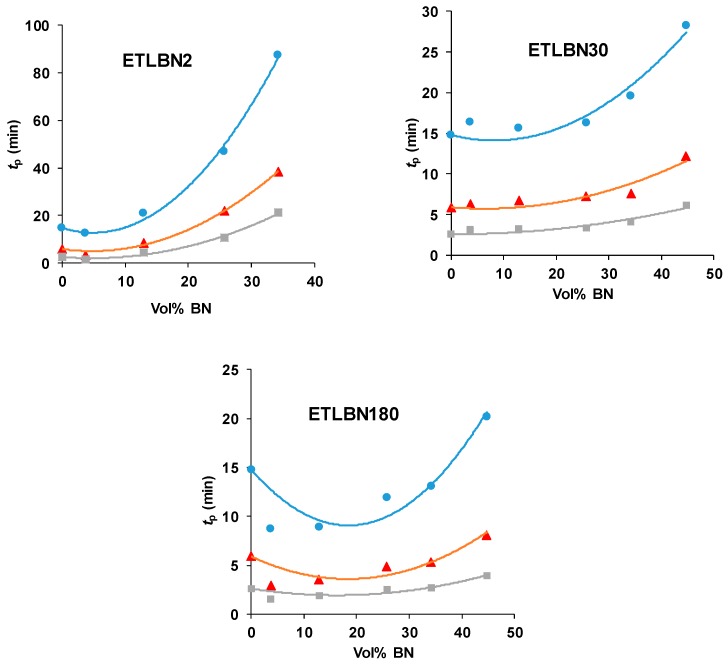
Time of exothermic peak during isothermal cure, *t*_p_, as a function of BN content for different platelet sizes, as indicated, and for different isothermal cure temperatures: 60 °C, blue circles; 70 °C, red triangles; 80 °C, grey squares.

The variation of the peak exotherm time, *t*_p_, with the BN content is shown in Figure 5 for the three BN platelet sizes and for the three isothermal cure temperatures. Here can be seen the tendency for the reaction first to accelerate, with a reduction in *t*_p_, and then to be slowed as the BN content increases. There is a systematic variation with the BN platelet size, whereby the peak time *t*_p_, for a given cure temperature and BN content, decreases as the BN platelet size increases, and dramatically so on going from 2 µm to 30 μm; this behavior mirrors that shown in Figure 3 for the non-isothermal cure.

**Figure 6:**
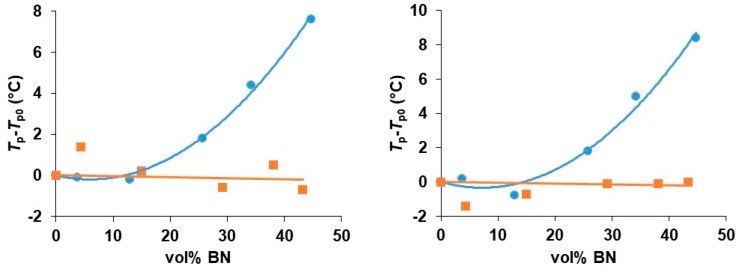
Peak temperature during non-isothermal cure relative to that for the unfilled system, *T*_p_−*T*_p0_, as a function of BN 30 µm content for the heating rates of 2 K/min (**left**) and 5 K/min (**right**). Epoxy-thiol system, blue circles; epoxy-diamine system, red squares.

In order to understand the role of the thiol cross-linking agent in the kinetics of the cure reaction, it is of interest to compare these results for the cure of these epoxy-BN composites with a thiol with those for the same epoxy-BN system cured with a diamine, Jeffamine D-420. This comparison has been made for just one of the BN platelet sizes (30 µm). The corresponding results for the non-isothermal cure of the EJBN30-*y* samples at 2 and at 5 K/min are shown together with the results for the ETLBN30-*y* samples at the same heating rates in Figure 6. It is evident that the peak temperature for the epoxy-diamine system is essentially independent of the BN filler content, in clear contrast to the behavior for the epoxy-thiol system. This was also reported earlier in the comparison of epoxy-thiol and epoxy-diamine systems filled with aluminium nitride, AlN [7].

**Figure 7:**
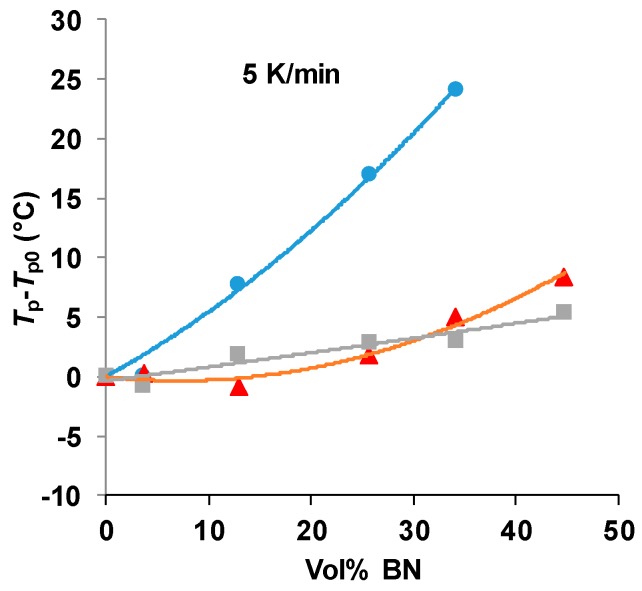
Peak temperature during non-isothermal cure of the epoxy-thiol system relative to that for the unfilled system, *T*_p_−*T*_p0_, as a function of BN content for the heating rate of 5 K/min. Effect of platelet size: 2 µm, blue circles; 30 µm, red triangles; 180 µm, grey squares.

There is clearly an influence of the BN filler on the cure kinetics of the epoxy-thiol system, though the fully cured epoxy network structure is unaffected, and this effect is related to the presence of the thiol as it is not observed for the epoxy-diamine system. In this context, the effect of the BN particle size is important. Figure 7 shows how the peak exotherm temperature in non-isothermal cure at 5 K/min depends on the platelet size. It can be seen that the greatest effect of retarding the reaction occurs for the smallest platelets, while there is very little difference between the effects of the 30 and 180 µm platelets. This behavior occurs also for the heating rates of 2 and 10 K/min, and the systematic effect of the particle size and of the filler content has been noted in earlier work [7,8].

Since this interaction between the epoxy matrix and the BN filler is fundamental in respect of the heat transfer by phonon transport in the composite material, this systematic behavior should be compared with the thermal conductivities of these epoxy-thiol composites filled with BN platelets of different sizes. First, however, it is of interest to consider the composition of the cured composites by means of thermogravimetric and density measurements.

### 3.2. Thermogravimetric Analysis (TGA)

**Figure 8:**
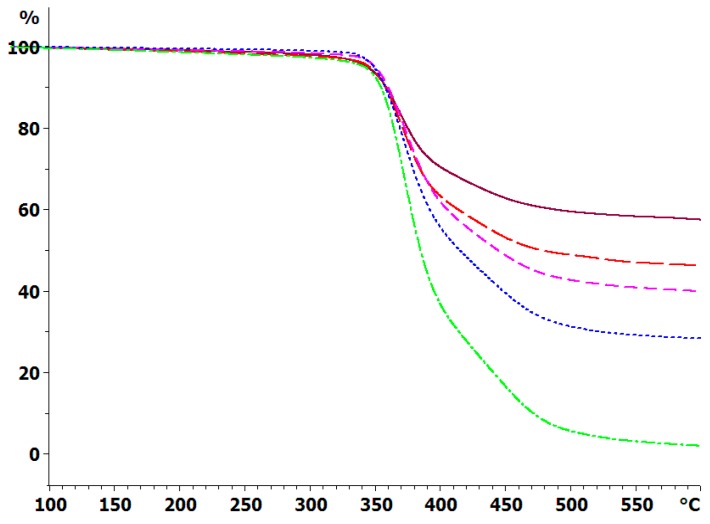
TGA curves of sample mass versus temperature for the fully cured ETLBN30-*y* system at 10 K/min in an inert atmosphere: dash-dotted, green, *y* = 0; dotted, blue, *y* = 30; short dash, pink, *y* = 50; long dash, red, *y* = 60; full line, purple, *y* = 70.

The thermal degradation of the cured systems has been determined by TGA under an inert atmosphere of dry nitrogen, and the weight loss at 10 K/min for the composites with the 30 μm BN platelets is shown in Figure 8; similar results were obtained for the composite samples with 2 and with 180 μm platelets, and for the heating rate of 2 K/min. In addition, the degradation behavior of the epoxy-thiol system without any filler (ETL) was also determined, and that of the BN particles alone were also studied by TGA, though these results are not shown in Figure 8. The degradation behavior is very similar for the composites fabricated with BN platelets of different sizes, as would be expected from our earlier observation that the epoxy network structure is independent of the BN platelet size and content. For each platelet size, the important difference between the various curves is the residual mass at 600 °C, which increases as the BN content increases.

**Figure 9:**
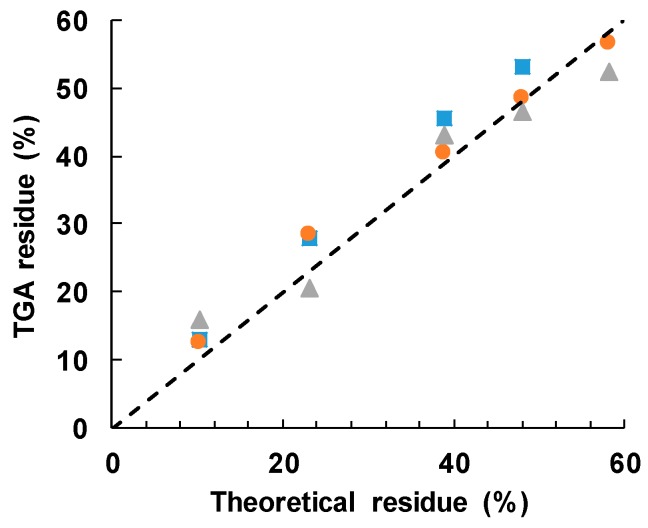
Measured TGA residue after degradation to 600 °C as a function of the theoretical value: 2 µm, blue squares; 30 µm, orange circles; 180 µm, grey triangles. The dashed line has a slope of unity.

The cured epoxy-thiol without any filler, ETL, leaves a residue of 4.5 ± 2.5%, while the BN particles alone leave a residue of 97 ± 2%. Taking a 60:40 mass ratio for the stoichiometric epoxy:thiol ratio, with 2 wt % LC-80 initiator, then the anticipated residues for the different BN contents can be calculated as: 10.2 ± 2.5% for ETLBN*x*-10; 23.2 ± 2.4% for ETLBN*x*-30; 38.9 ± 2.3% for ETLBN*x*-50; 48.0 ± 2.2% for ETLBN*x*-60; 58.2 ± 2.2% for ETLBN*x*-70, independently of the size, *x*, of the platelets. The average values obtained by TGA for the two different heating rates of 2 and 10 K/min are plotted as a function of these theoretical values in Figure 9. It can be seen that the correlation is very good, confirming thus the BN contents of the different composite samples as well as supporting the conclusion that the epoxy network structure is independent of the filler size and content.

### 3.3. Density Measurements

**Figure 10:**
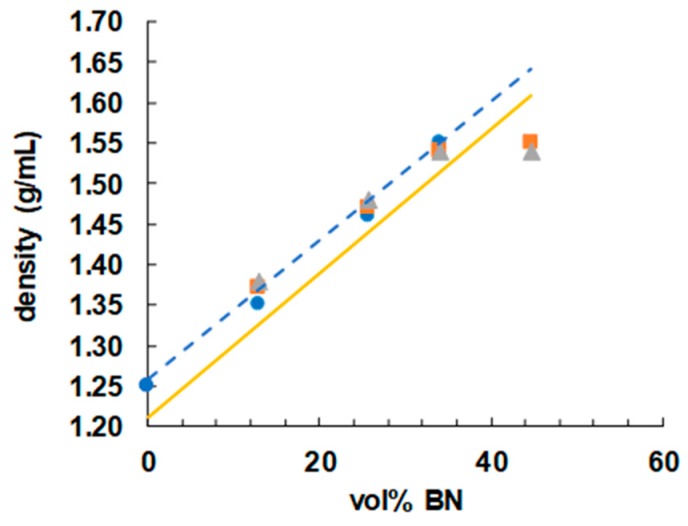
Density as a function of vol % BN for epoxy-thiol samples with different BN platelet sizes: 2 µm, blue circles; 30 µm, orange squares; 180 µm, grey triangles.

In order to achieve a high thermal conductivity in these samples, it is important that they be free from voids. A good way to check for the absence of voids in the cured samples is by means of density measurements, and the results are shown in Figure 10 as a function of BN vol %. If it is assumed that the volumes of the components that make up the epoxy-thiol-BN composite are additive, then, using the densities of the various components (epoxy 1.17; thiol 1.28, BN 2.1 g/mL), an approximate calculation of the anticipated density of any of the compositions listed in Table 1 can be made. The dependence of this calculated density on the BN content is indicated in Figure 10 by the full yellow line, where it can be seen that, apart from the results for the highest BN content of 44.7 vol %, the measured densities are all higher than the predicted values. The reason for this is that, during the epoxy-thiol reaction, the volumes are not in fact additive, but there is typically a 4% volume shrinkage on cure. When this shrinkage is taken into account, the predicted densities follow the dashed blue trend-line shown in Figure 10, in good agreement with the measured values except for those at 44.7 vol %. There is probably a significant void content in this composite with the highest BN content; the uncured mixture was a very stiff paste, and the degassing procedure would not eliminate any air bubbles introduced during the mixing process. This is likely to have an impact on the thermal conductivity, discussed shortly.

### 3.4. Thermal Conductivity

**Figure 11:**
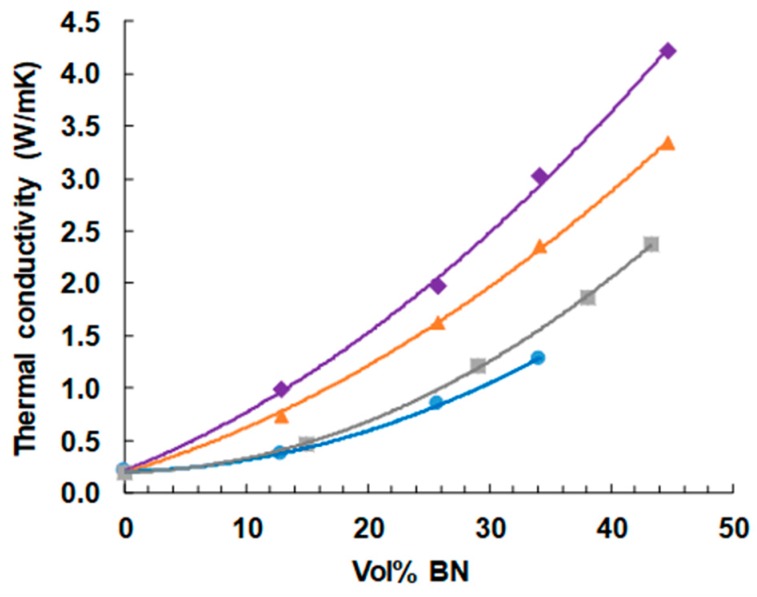
Thermal conductivity of ETLBN and EJBN samples as a function of the BN content: ETLBN2, blue circles; EJBN30, grey squares; ETLBN30, orange triangles; ETLBN180, purple diamonds.

The dependence of thermal conductivity on BN content is shown in Figure 11. As expected, and as has been observed in the large majority of studies of such systems [reference 8 gives a compilation], the thermal conductivity increases with BN content, and more so the greater is the BN content, giving an upward curvature to the dependence. The highest vol % of 44.7% represents the approximate limit that can be achieved with the simple mixing procedures adopted here, as the mixture becomes a very stiff paste at these BN contents, and is intractable for higher concentrations. Indeed, the vol % of 44.7% cannot be achieved for the samples with 2 μm platelets, as explained in an earlier section.

It can also be seen very clearly that the thermal conductivity increases systematically with increasing BN platelet size. In this study, we have not made use of any surface treatment of the BN particles or of any coupling agent to improve the contact between particles and matrix. Furthermore, the BN particles all have the same shape (platelets), as well as all being from the same supplier (Saint Gobain). There can therefore be no doubt that increasing BN particle size results in an increase in the thermal conductivity when composites with the same filler content are compared. This confirms what the majority of studies have suggested, but which until now has been unclear as a consequence of the possible influence of the other parameters, which have been eliminated in the present study.

The increase of thermal conductivity with BN particle size can be understood from a consideration of the interface between particles and matrix, and of the geometry of the particles. In view of the very high value of thermal conductivity of hexagonal BN (600 W/mK parallel to the basal plane; 30 W/mK perpendicular to the basal plane), the fact that more limited values are obtained for epoxy-BN composites in general is evidently in part a consequence of the interface between particles and matrix, which presents a resistance to heat flow. Indeed, this is the reason why coupling agents and surface treatments are often introduced into the composite preparation procedure, though not always with a significant improvement in the thermal conductivity. For a given BN content, the larger is the particle size, the smaller is the interfacial area, and hence increased particle size is clearly beneficial. However, if the platelets are considered to be hexagonal with edge size d/2 and thickness *t*, then the ratio of area:volume is *A*/*V* = 2/*t* + 8/*d*√3. With *t* = 0.2 μm this gives values of *A*/*V* of 12.3, 10.2 and 10.0 μm^−1^ for the 2, 30 and 180 μm platelets, respectively; with *t* = 1.0 μm the corresponding values of *A*/*V* are 4.3, 2.2 and 2.0. The increase of 20 to 30% in the thermal conductivity between the samples with 30 and 180 μm platelets is difficult to reconcile with the much smaller increase in the corresponding area:volume ratio.

It is in this respect that the geometry of the particles is believed to play an important part, and in particular the anisotropy of the thermal conductivity. The larger platelets provide significantly more continuity in the parallel direction, with a thermal conductivity of 600 W/mK, than do the smaller platelets, and this additional factor results in the highest thermal conductivities of the 180 μm composites.

In fact, these composites with BN platelets of 180 μm, and also those with 30 µm platelets to a certain extent attain values that are significantly larger than most published values for the same BN content [8]. We believe that the reason for this is associated with the improved interface between matrix and particles as a consequence of the use of thiol as the cross-linking agent. This improvement results from the Lewis acid-base interaction between the sulphur of the thiol and the boron of the particles. In order to investigate this interpretation, epoxy-BN composite samples with the 30 µm platelets (EJBN30-*y*) were prepared in which the cross-linking agent was a diamine rather than the thiol. The resulting thermal conductivities as a function of the BN content are included in Figure 11, where it can be seen that they fall significantly below the corresponding values for the epoxy-BN composites cross-linked with thiol. The explanation for this is the matrix-filler interface is enhanced when the sulphur-containing thiol is used.

This distinction between the behaviors of the epoxy-BN composite system filled with 30 µm BN platelets and cured with thiol on the one hand and with diamine on the other was seen earlier in the cure kinetics monitored by DSC, shown in Figure 6. For the composites cured with thiol there is a strong and systematic dependence of the peak temperature on BN content, whereas for the diamine the peak temperature is essentially independent of the filler content. This confirms that there is an interaction between the epoxy-thiol matrix and the BN particles which influences both the cure kinetics and the matrix-filler interface, this latter leading to an increased thermal conductivity, whereas there is no such interaction when epoxy-diamine is used for the matrix. None of the results published by other authors and discussed here made use of thiol as a cross-linking agent, and we attribute the higher thermal conductivity values reported here to the advantageous interactions between the thiol and the filler particles.

### 3.5. Scanning Electron Microscopy

**Figure 12:**
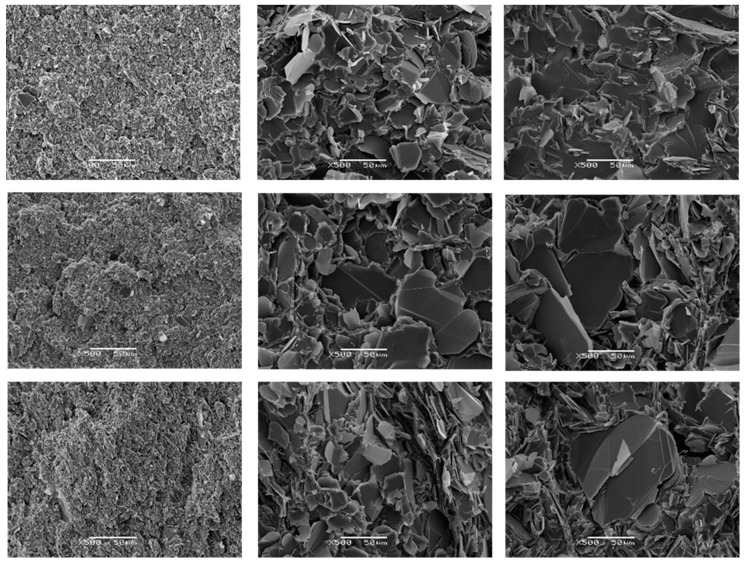
SEM micrographs of epoxy-thiol composites. Left hand column, top to bottom: ETLBN2 -30, -50, -60; middle column, top to bottom: ETLBN30 -30, -50, -60; right hand column, top to bottom: ETLBN180 -30, -50, -60. Magnification 500×, scale bar 50 μm.

The nature of the interface between BN particles and epoxy matrix has been investigated by examining the fracture surfaces of the cured samples by Scanning Electron Microscopy (SEM). The fracture surfaces of composite samples with different BN platelet sizes *x*, where *x* is 2, 30 and 180 µm, and different contents (ETLBN*x*-30, 12.9; ETLBN*x*-50, 25.7; ETLBN*x*-60, 34.2 vol %) are shown in Figure 12.

**Figure 13:**
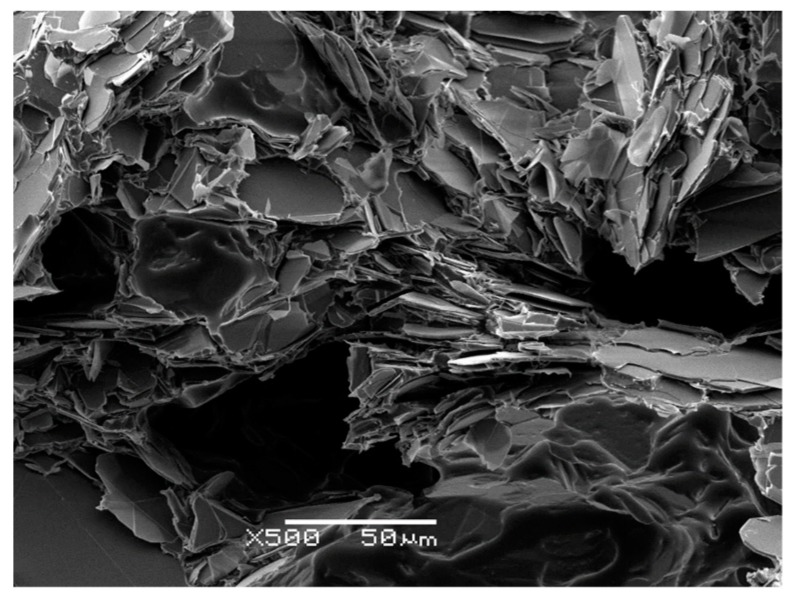
SEM micrograph of an epoxy-composite, EJBN30-60. Magnification 500×, scale bar 50 μm.

It can be seen, particularly for the larger platelet sizes, that there are many BN particles with no epoxy matrix adhering to the flat and smooth surfaces. This is an indication that the interface between the surfaces of the BN particles and the epoxy-thiol matrix is relatively poor, since the fracture has simply separated the matrix and filler along this interface. Nevertheless, this interface is better for the epoxy-thiol composites in comparison with the epoxy-amine composites, as can be seen by a comparison with the fracture surface of a corresponding epoxy-amine composite with a similar vol % of BN, shown in Figure 13. In this Figure, it can be seen that there are distinct resin-rich regions, indicative of a less homogeneous mixture, there is significant porosity, and the separation between the platelets and their surrounding epoxy matrix appears larger for the epoxy-amine composites in comparison with the epoxy-thiol composites. This is consistent with our hypothesis that the epoxy-thiol system gives an improved interaction between matrix and filler.

In spite of the significant increase in thermal conductivity achieved here by the addition of BN particles, this relatively poor interface, which represents a barrier to phonon transport, is one of the main reasons why the thermal conductivity of these composites does not achieve higher values. Many attempts have been made to improve this interface, for example by surface treatment of the BN particles or the use of coupling agents, with mixed success, as has been discussed briefly above. Likewise, the use of high pressure during the fabrication of these composites, which might be expected to improve the interface, has also been reported [10,14,15,17,26,27,28], though no direct comparison to observe the effect of pressure has been shown; this is currently under investigation in our laboratory, and will be reported in due course.

## 4. Conclusions

The thermal conductivity of epoxy-thiol composites filled with boron nitride, BN, particles in the form of platelets has been shown to increase with BN content in the usual way. However, for the same vol% of BN, the thermal conductivity increases with increasing BN particle size; this result clearly demonstrates the effect of the size of the particles, independent of other factors such as particle shape and surface treatment. The thermal conductivities of these epoxy-thiol-BN composites are higher than many reported in the literature, and this is attributed to an improved matrix-particle interface as a consequence of the Lewis acid-base interaction between the thiol and the BN particles. This interpretation is supported by similar experiments with an epoxy-diamine system, for which the thermal conductivity for the same BN content and the same BN platelet size is significantly lower. The cure kinetics likewise shows a difference between the epoxy-thiol and epoxy-diamine composites. For the former, there is a systematic dependence of the cure kinetics on the BN content, which mirrors the thermal conductivity results, whereas for the latter the cure kinetics is essentially independent of the BN content. This result again supports the conclusion that the use of thiol as a cross-linking agent enhances the matrix-filler interface, and hence increases the thermal conductivity.

## Figures and Tables

**Table 1:** Composition by weight of epoxy-thiol samples filled with BN particles with size *x* = 2, 30, 180 µm.

Sample	Epoxy	BN	Thiol	LC-80
ETL	100	0	67	2.0
ETLBN*x*-10	90	10	60.3	1.8
ETLBN*x*-30	70	30	47	1.4
ETLBN*x*-50	50	50	33.5	1.0
ETLBN*x*-60	40	60	27	0.8
ETLBN*x*-70	30	70	20	0.6

## References

[1] Ventec International Group. http://www.ventec-group.com/products/tec-thermal-thermal-management-ims/vt-4b3/datasheet/.

[2] Insulated Metal Substrate Copper Clads & Pre-Preg. https://www.aitechnology.com/products/insulated-metal-substrates/thermclads/.

[3] Boron Nitride, Thermal Properties. http://www.ioffe.ru/SVA/NSM/Semicond/BN/thermal.html.

[4] Rumyantsev S.L., Levinshtein M.E., Jackson A.D., Mohammmad S.N., Harris G.L., Spencer M.G., Shur M.S., Levinshtein M.E., Rumyantsev S.L., Shur M.S. (2001). Boron nitride (BN). Properties of Advanced Semiconductor Materials: GaN, AlN, InN, BN, SiC, SiGe.

[5] Chen H., Ginzburg V.V., Yang J., Yang Y., Liu W., Huang Y., Du L., Chen B. (2016). Thermal conductivity of polymer-based composites: Fundamentals and applications. Prog. Polym. Sci..

[6] Burger N., Laachachi A., Ferriol M., Lutz M., Toniazzo V., Ruch D. (2016). Review of thermal conductivity in composites: Mechanisms, parameters and theory. Prog. Polym. Sci..

[7] Hutchinson J.M., Román F., Cortés P., Calventus Y. (2017). Epoxy composites filled with boron nitride and aluminium nitride for improved thermal conductivity. Polimery.

[8] Hutchinson J.M., Román F., Folch A. (2018). Epoxy-thiol systems filled with boron nitride for high thermal conductivity applications. Polymers.

[9] Yung K.C., Wang J., Yue T.M. (2008). Thermal management for boron nitride filled metal core printed circuit board. J. Comp. Mater..

[10] Hong J.-P., Yoon S.-W., Hwang T., Oh J.-S., Hong S.-C., Leeb Y., Nam J.-D. (2012). High thermal conductivity epoxy composites with bimodal distribution of aluminum nitride and boron nitride fillers. Thermochim. Acta.

[11] Kim K., Kim M., Hwang Y., Kim J. (2014). Chemically modified boron nitride epoxy terminated dimethylsiloxane composite for improving the thermal conductivity. Ceram. Int..

[12] Huang L., Zhu P., Li G., Zhou F., Lu D., Sun R., Wong C. (2015). Spherical and flake-like BN filled epoxy composites: Morphological effect on the thermal conductivity, thermo-mechanical and dielectric properties. J. Mater. Sci. Mater. Electron..

[13] Gaska K., Rybak A., Kapusta C., Sekula R., Siwek A. (2015). Enhanced thermal conductivity of epoxy-matrix composites with hybrid fillers. Polym. Adv. Technol..

[14] Chung S.-L., Lin J.-S. (2016). Thermal conductivity of epoxy resin composites filled with combustion synthesized h-BN particles. Molecules.

[15] Hong J.-P., Yoon S.-W., Hwang T.-S., Lee Y.-K., Won S.-H., Nam J.-D. (2010). Interphase control of boron nitride/epoxy composites for high thermal conductivity. Korea-Aust. Rheol. J..

[16] Yung K.C., Liem H., Choy H.S. (2013). Prerequisite for maximizing thermal conductivity of epoxy laminate using filler. J. Mater. Sci. Mater. Electron..

[17] Wattanakul K., Manuspiya H., Yanumet N. (2011). Thermal conductivity and mechanical properties of BN-filled epoxy composite: Effects of filler content, mixing conditions, and BN agglomerate size. J. Comp. Mater..

[18] Zhou W.Y., Qi S.H., Zhao H.Z., Liu N.L. (2007). Thermally conductive silicone rubber reinforced with boron nitride particle. Polym. Comp..

[19] Permal A., Devarajan M., Hung H.L., Zahner T., Lacey D., Ibrahim K. (2016). Thermal and mechanical properties of epoxy composite filled with binary particle system of polygonal aluminum oxide and boron nitride platelets. J. Mater. Sci..

[20] Brändle A., Khan A. (2012). Thiol-epoxy “click” polymerization: Efficient construction of reactive and functional polymers. Polym. Chem..

[21] Belmonte A., Guzman D., Fernández-Francos X., De La Flor S. (2015). Effect of the network structure and programming temperature on the shape-memory response of thiol-epoxy “click” systems. Polymers.

[22] CarboTherm Thermal Management Fillers. https://www.bn.saint-gobain.com/sites/imdf.bn.com/files/carbotherm-bn-thermal-fillers-ds.pdf.

[23] Tanaka T., Wang Z., Iizuka T., Kozako M., Okhi Y. High thermal conductivity epoxy/BN composites with sufficient dielectric breakdown strength. Proceedings of the IEEE 2011 Annual Report Conference on Electrical Insulation and Dielectric Phenomena.

[24] Kim K., Kim J. (2014). Fabrication of thermally conductive composite with surface modified boron nitride by epoxy wetting method. Ceram. Int..

[25] Hammerschmidt U., Meier V. (2006). New Transient Hot-Bridge sensor to measure thermal conductivity, thermal diffusivity, and volumetric specific heat. Int. J. Thermophys..

[26] Wattanakul K., Manuspiya H., Yanumet N. (2010). The adsorption of cationic surfactants on BN surface: Its effects on the thermal conductivity and mechanical properties of BN-epoxy composite. Colloids Surf. A Physicochem. Eng. Aspects.

[27] Wattanakul K., Manuspiya H., Yanumet N. (2011). Effective surface treatments for enhancing the thermal conductivity of BN-filled epoxy composite. J. Appl. Polym. Sci..

[28] Hu J., Huang Y., Zeng X., Li Q., Ren L., Sun R., Xu J.-B., Wong C.-P. (2018). Polymer composite with enhanced thermal conductivity and mechanical strength through orientation manipulating of BN. Comp. Sci. Technol..

